# Supplementary material for: From noise to models to numbers: Evaluating negative binomial models and parameter estimations in single-cell RNA-seq
Source: PLoS Comput Biol. 2026 Mar 16;22(3):e1014014. doi: 10.1371/journal.pcbi.1014014 (PMC13046287; doi:10.1371/journal.pcbi.1014014)
Supplement: S1 Appendix — Supplemental figures include the relative error of aeBIC compared with the expectation of BIC for Poisson and negative binomial (NB) distributions across sample sizes and selected parameter sets (Fig A), the relative error between the minimum cross-entropy determined by MLE and moment matching for the NB approximation under different transcript capture probabilities (Fig B), phase diagrams showing aeBIC-based model selection among technical-noise corrected telegraph/NB/Poisson models for different capture-probability distributions (Fig C), a simulated scRNA-seq workflow with technical noise and downstream comparisons of burst-frequency/burst-size estimation error and ranking consistency (Fig D), recalculated versions of the statistics in Fig D under alternative capture distributions and switching-rate regimes (Nσ=10 and 20) (Fig E), an evaluation showing that Hessian eigenvalue ratios are not reliable indicators for model selection compared with aeBIC boundaries (Fig F), and an aeBIC phase diagram for telegraph/NB/Poisson model selection at large sample size (nc=106) (Fig G). Supplemental tables include the parameter sets used in Fig 4b and Fig A(a,b) (Table A), and a comparison of model selection by aeBIC and BIC for the simulations in Fig 4c with nc=100 cells (Table B). References are provided at the end of the appendix. (PDF) [file pcbi.1014014.s001.pdf]

Supplementary Figures

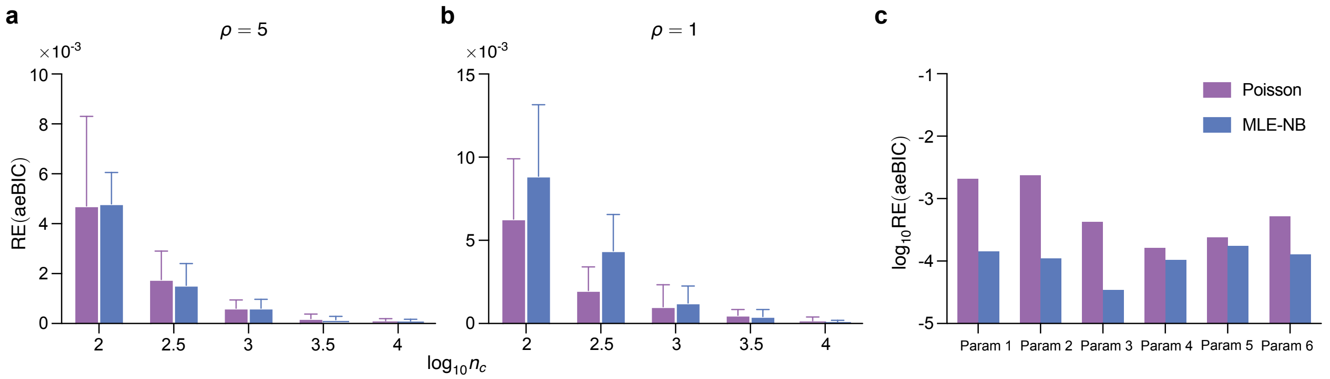

Fig A: (a)(b) The relative error of aeBIC compared to the expectation of BIC ($\mathbf{E}$[BIC]) for two distributions (Poisson and NB) as a function of sample size n_c for 10 parameter sets (see Table A for the values of N_σ and f_{on}) with (a) $\rho = 5$ and (b) $\rho = 1$. Error bars show the standard error of the mean. (c) Relative error of aeBIC with respect to $\mathbf{E}$[BIC] for Poisson and NB distributions, evaluated for 6 parameter sets sampled from the plot corresponding to $n_c = 10^4$, $\rho = 15$ and $p_{\text{cap}} \sim \text{Beta}(60, 140)$ in Fig. 6c. The parameter sets are: $f_{\text{on}} = 0.8, N_\sigma = 3.8$ (Param 1), $f_{\text{on}} = 0.88, N_\sigma = 1.5$ (Param 2), $f_{\text{on}} = 0.5, N_\sigma = 38$ (Param 3), $f_{\text{on}} = 0.96, N_\sigma = 758.6$ (Param 4), $f_{\text{on}} = 0.7, N_\sigma = 380.2$ (Param 5), $f_{\text{on}} = 0.1, N_\sigma = 631$ (Param 6).

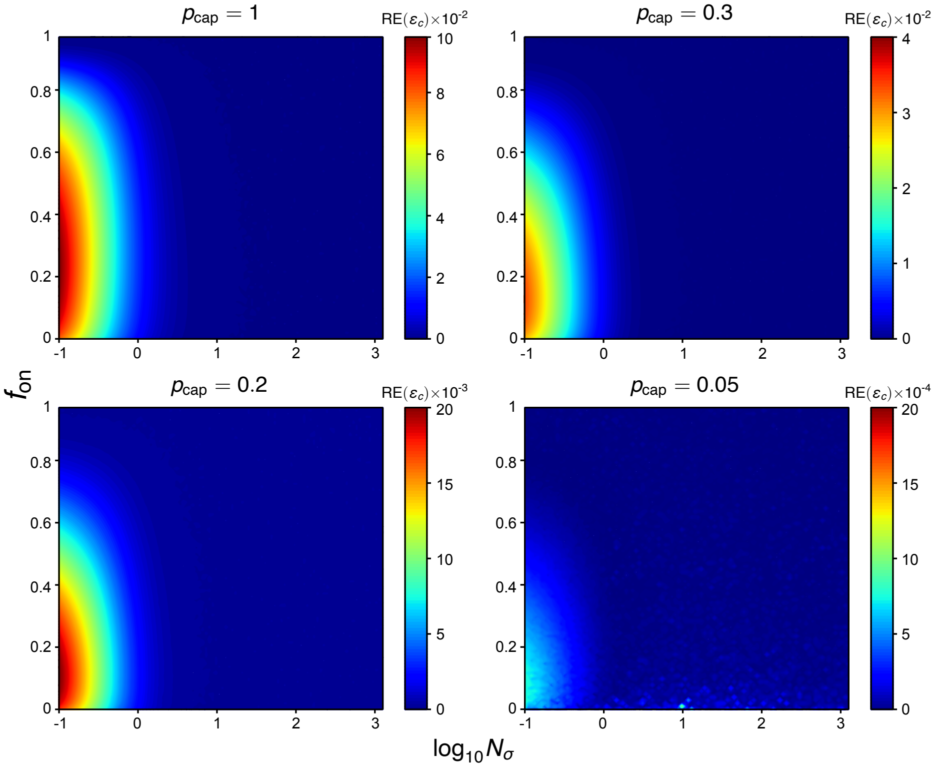

Fig B: The relative error between the minimum cross-entropy determined by MLE and moment matching. The cross-entropy is that between the NB distribution as the proposed model ($P_{\mathcal{M}}(n|\theta)$) and the telegraph model distribution Eq. (1) with $\rho \to \rho p_{\rm cap}$ as the ground-truth distribution ($P_{\mathcal{G}}(n)$), assuming all cells have an identical mRNA capture probability $p_{\rm cap}$. The minimum cross-entropy using MLE is obtained by finding the two parameters of the NB distribution which minimize Eq. (11). The minimum cross-entropy using moment-matching is obtained by directly evaluating Eq. (11) using the effective NB distribution (Eq. (6)) with $\rho \to \rho p_{\rm cap}$. As the heat maps show, the relative error between the two minimum cross-entropies is very small, regardless of the values of $p_{\rm cap}$, $f_{\rm on}$ and N_σ. Note that ρ is in all cases fixed to 15.

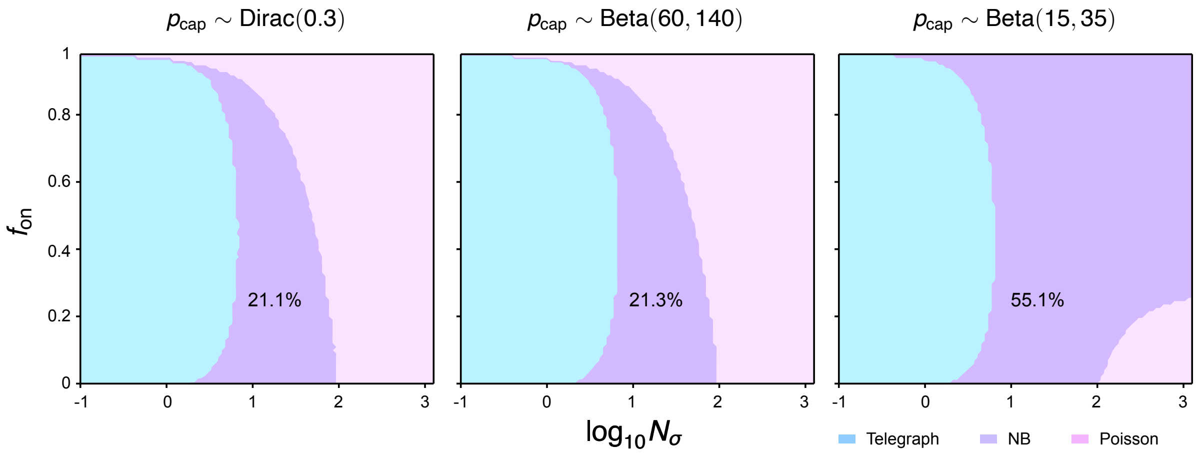

Fig C: Phase diagram showing the regions of parameter space where the technical-noise corrected telegraph, NB and Poisson models are selected as the optimal models by the aeBIC, given that the ground-truth mRNA distribution is that of the corrected telegraph model, i.e. the telegraph model with $p_{\rm cap}$ sampled from Dirac(0.3), Beta(60, 140) and Beta(15, 35) — these distributions have mean 0.3 and CV = 0, 0.11 and 0.21, respectively. Here N_σ is the sum of gene-state switching rates normalised by the degradation rate of mRNA, and $f_{\rm on}$ is the fraction time spent in the active state. The fraction of the total parameter space occupied by the region where the corrected NB distribution is optimally selected is shown on the plots. Note that the transcription rate is fixed to $\rho = 15$. The maximum mean number of transcripts in the phase plots is 4.5. The sample size is fixed to $n_c = 10^4$.

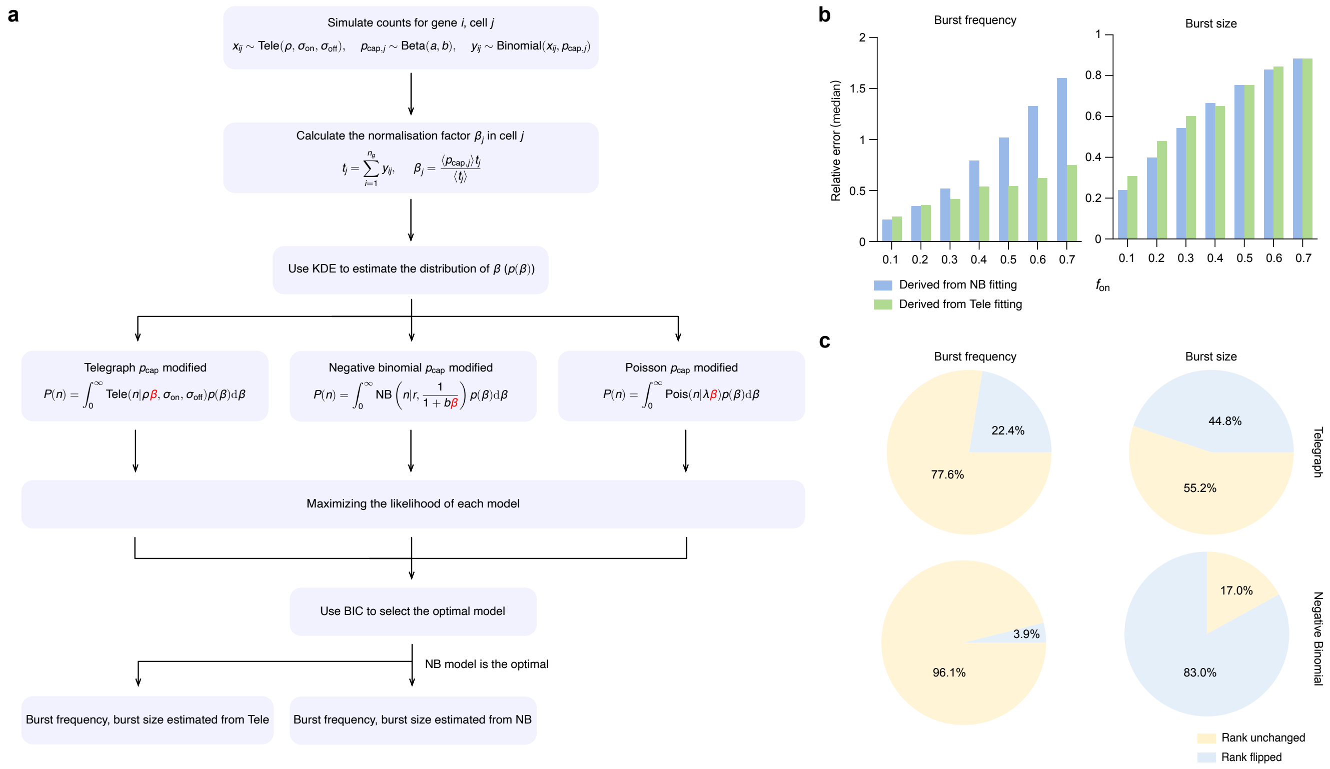

Fig D: (a) Flowchart of the procedure described in Section 4.6 which simulates the workflow of a scRNA-seq experiment. A synthetic scRNA-seq dataset (1000 cells and 2800 genes) is simulated — technical noise is account for by sampling the capture probability for cell j ($p_{\text{cap},j}$) from a Beta(15, 35) distribution. The data is then used to perform MLE-based parameter inference and BIC-based model selection. Notably, the latter is done without exact knowledge of the capture rate distribution, which mimics the real scenario. Only knowledge of the mean capture probability p_{cap} is assumed which can be estimated using spike-in controls [1] or by comparison of scRNA-seq data with smFISH data [2]. (b) Plots of the median relative error of the estimates of the burst frequency and burst size as a function of the true fraction of time spent in the active state f_{on} by genes. The estimates are for those genes whose distribution of counts per cell is best fit by an NB distribution (after correcting for technical noise). The estimates are obtained by maximizing the likelihood of the technical-noise corrected NB and telegraph models (blue and green bars, respectively). (c) 10^3 sets of two pairs of burst size and burst frequency estimates are randomly selected. In each set, genes are ranked by the magnitude of the burst frequency and separately by the magnitude of the burst size. The piecharts show the percentage of rankings that are flipped compared to the true ranking. The rankings are separately done using the parameter estimates obtained by maximizing the likelihood of the technical-noise corrected NB and telegraph models (piecharts in the bottom and top rows, respectively).

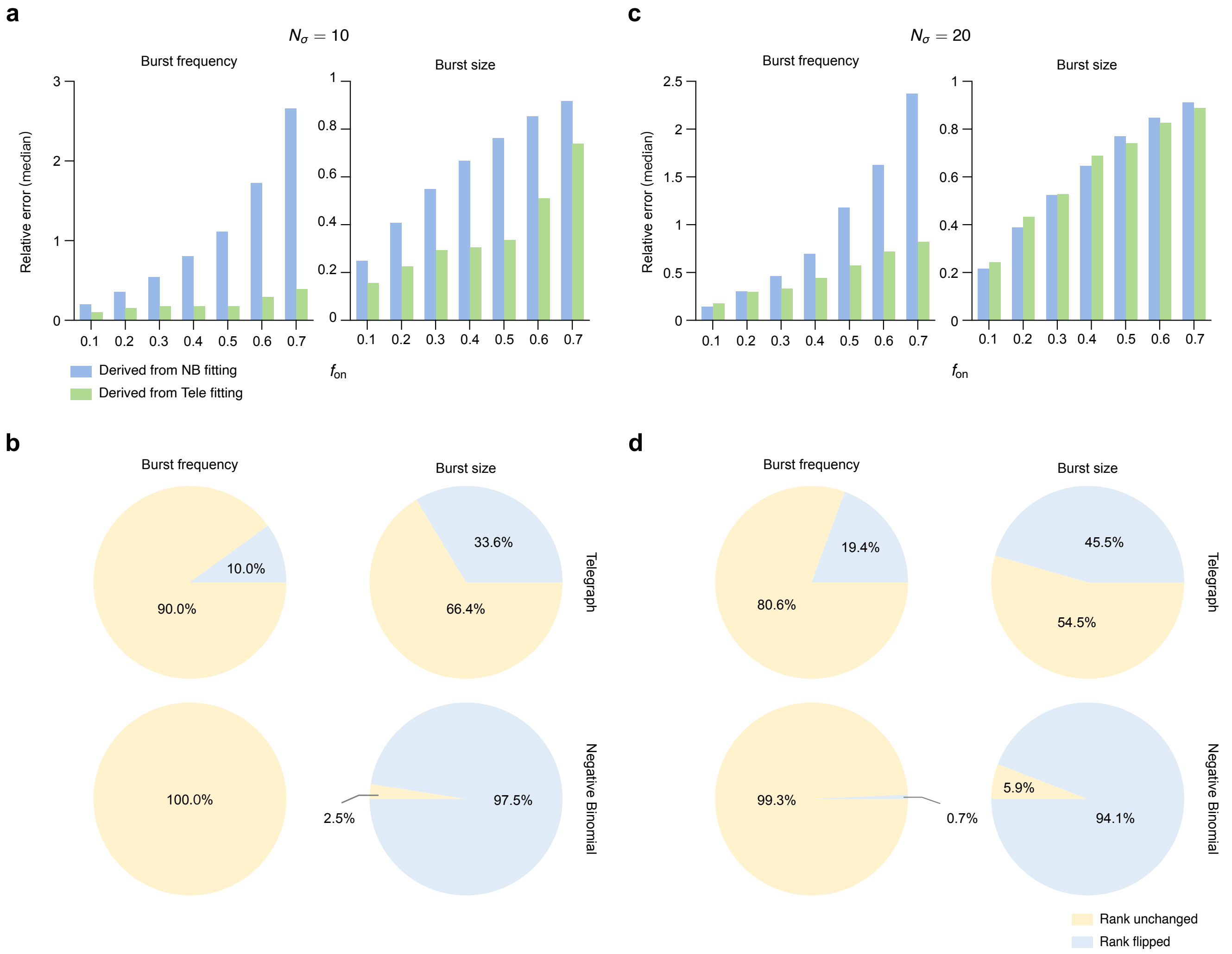

Fig E: (a)(b)Recalculation of the statistics shown in Fig. D(b)(c) using the procedure described in Section 4.6 with the difference that the number of cells is $n_c = 10^4$ and the transcript capture distribution is sampled from Beta$(60, 140)$. Note that $N_\sigma = 10$. (b)(d) Recalculation of the statistics shown in (a) and (b) with the difference that $N_\sigma = 20$. Note that 10^3 sets of two pairs of burst size and burst frequency estimates were used to compute the piecharts for the gene rankings in (b) and (d).

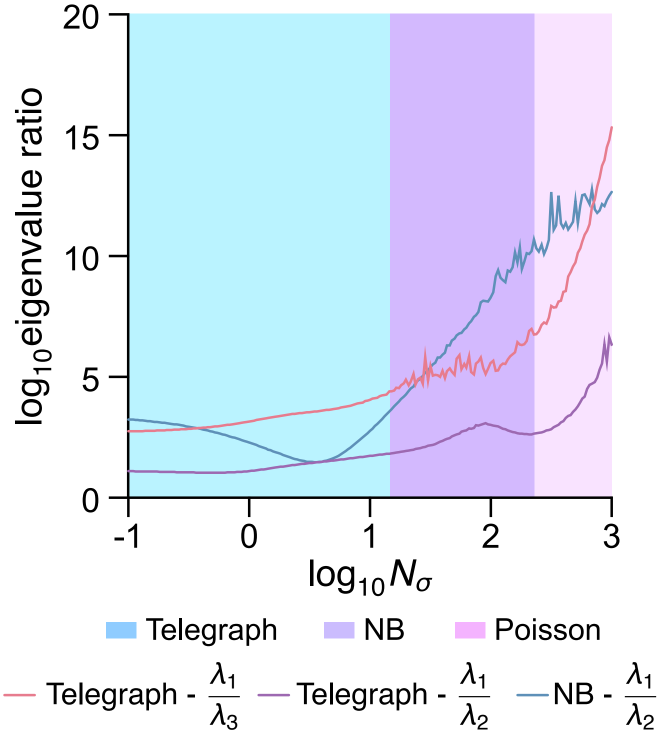

Fig F: Eigenvalues of the Hessian matrix of the log-likelihood are not reliable indicators for model selection. We varied N_σ from 0.1 to 10^3, while fixing $\rho = 15$, $f_{\text{on}} = 0.3$, and the number of cells at 10^4. The corresponding values of σ_{on} and σ_{off} were determined by Eq. (7) in the main text. For fair comparison with the aeBIC method, data were generated directly from Eq. (1) using these parameters. The log-likelihood was defined as $\mathcal{L}_\theta = n_c \sum_n P(n) \ln P_\theta(n)$, where $P_\theta(n)$ is the probability given by either the telegraph or NB model with parameters θ. Under this setup, the telegraph model recovers the generative parameters exactly, while the NB parameters follow Eq. (6). We then computed the eigenvalues of the Hessian matrix at the MLEs for each model: three eigenvalues for the telegraph model and two for the NB model, all negative. Denoting the ordered eigenvalues as $\lambda_1, \lambda_2, \lambda_3$, we examined the ratios $-\lambda_1/\lambda_2$ and $-\lambda_1/\lambda_3$ for the telegraph model, and $-\lambda_1/\lambda_2$ for the NB model, as N_σ increased. These ratios (lines) gradually become infinite as N_σ grows, reflecting model degeneration. In contrast, the aeBIC method yields clear model selection boundaries (shaded areas). Together, these results indicate that Hessian eigenvalues are not reliable indicators for model selection.

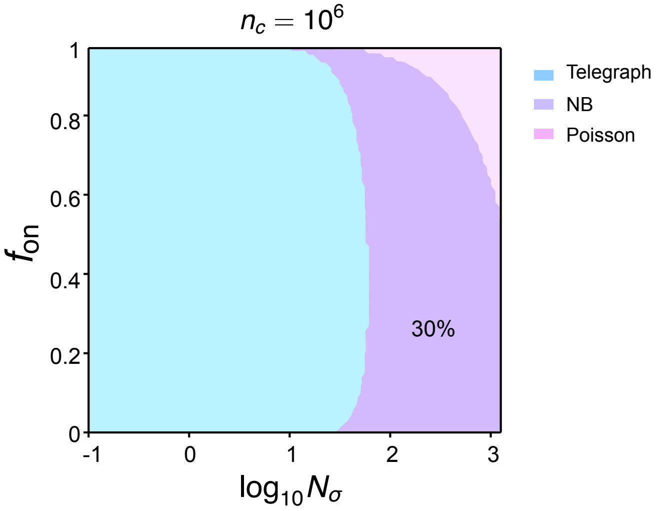

Fig G: Phase diagram showing the regions of parameter space where the telegraph, NB and Poisson distributions are selected as optimal by the aeBIC, given that the ground-truth mRNA distribution is that of the telegraph model, for a sample size of $n_c = 10^6$. Here N_σ is the sum of gene-state switching rates normalised by the degradation rate of mRNA, and f_{on} is the fraction of time spent in the active state. The fraction of the total parameter space occupied by the region where the NB distribution is optimally selected is shown on the plot. Note that the transcription rate is fixed to $\rho = 15$, which implies that the maximum mean number of transcripts in the phase diagram is 15.

Table A: Parameter sets used in Fig. 4b and Fig. Aa,b.

	Parameters	
	f_{on}	N_σ
Set 1	0.1	1
Set 2	0.4	10
Set 3	0.2	10
Set 4	0.7	100
Set 5	0.5	1
Set 6	0.9	0.1
Set 7	0.3	1000
Set 8	0.8	100
Set 9	0.6	0.1
Set 10	0.6	1000

Table B: Comparison of the models selected by the aeBIC score and the BIC score for Fig. 4c with $n_c = 100$ cells. The minimum aeBIC score directly leads to a unique model that is shown in the first column. The BIC scores for the telegraph, NB and Poisson models are computed using MLE for each of 100 independent samples of size n_c generated by sampling from the Beta-Poisson formulation of the telegraph model Eq. (2) for the parameters shown in the 2nd and 3rd columns and $\rho = 15$; for each sample, the model with the smallest BIC score is selected (% of models selected are shown in the last 3 columns).

Region	Parameters		Percentage of selected models by BIC		
	f_{on}	N_σ	Telegraph	Negative Binomial	Poisson
Tele	0.8	0.3	100%	0%	0%
	0.5	0.1	100%	0%	0%
NB	0.4	10	7%	88%	5%
	0.2	7	7%	93%	0%
Pois	0.9	100	0%	1%	99%
	0.1	400	0%	1%	99%

References

[1] V. Svensson, "Droplet scRNA-seq is not zero-inflated," *Nature Biotechnology*, 38(2): 147–150, 2020.

[2] W. Tang, F. Bertaux, P. Thomas, C. Stefanelli, M. Saint, S. Marguerat, and V. Shahrezaei, "Baynorm: Bayesian gene expression recovery, imputation and normalization for single-cell RNA-sequencing data," *Bioinformatics*, 36(4): 1174–1181, 2020.
